# Application of optical coherence tomography and keratograph in the measurements of lower lid margin thickness

**DOI:** 10.1007/s00417-023-05990-w

**Published:** 2023-03-02

**Authors:** Da-Hu Wang, Jian-Cen Tang, Xiao-Jun Hao, Yin-Jian Zhang, Xin-Quan Liu

**Affiliations:** 1 Department of Ophthalmology, LongHua Hospital Affiliated to Shanghai University of Traditional Chinese Medicine, Shanghai, China; 2 Eye Research Institute, Longhua Hospital Affiliated to Shanghai University of Traditional Chinese Medicine, Shanghai, China; 3 Department of Ophthalmology, Shanghai East Hospital, Tongji University School of Medicine, Shanghai, China; 4 Department of Ophthalmology, Shanghai Punan Hospital, Shanghai, China

**Keywords:** Lid margin thickness, Optical coherence tomography, Keratograph, Tear meniscus height, Meibomian gland dysfunction, Dry eye disease

## Abstract

**Purpose:**

This study aims to investigate the applicability of lower lid margin thickness (LLMT) measurements in adults with and without meibomian gland dysfunction (MGD) by optical coherence tomography (OCT) and keratograph.

**Methods:**

This is a cross-sectional, observational study. A hundred and eight volunteers aged 20 to 79, including 68 MGD patients and 40 normal subjects, were recruited. Using OCT and keratograph to measure the LLMT from the posterior lash line to anterior edge or outer edge of the tear meniscus was separately performed two times by the same person.

**Results:**

The mean age of normal and MGD subjects was 50.5 ± 14.2 years and 55.8 ± 15.5 years, respectively. The LLMT with OCT and keratograph in MGD patients was significantly greater than that in normal subjects (1.06 ± 0.27 and 1.03 ± 0.25 mm vs. 0.90 ± 0.20 and 0.86 ± 0.16 mm, respectively). In both normal and MGD subjects, the tear meniscus height and LLMT with OCT were both greater than that with keratograph (*P* < 0.05), and intraclass correlation coefficient (ICC) demonstrated a good agreement in the LLMT measurements between two devices (ICC = 0.83 and 0.79, respectively). Additionally, the LLMT in MGD patients was appeared to be positively correlated with meiboscore (*r*_s_ = 0.37, *P* = 0.002).

**Conclusions:**

The OCT and keratograph were two reliable tools in the LLMT measurements, which may have potential applications for diagnosis and evaluation of MGD. Furthermore, we found that the LLMT measured by OCT was greater than that measured by keratograph.


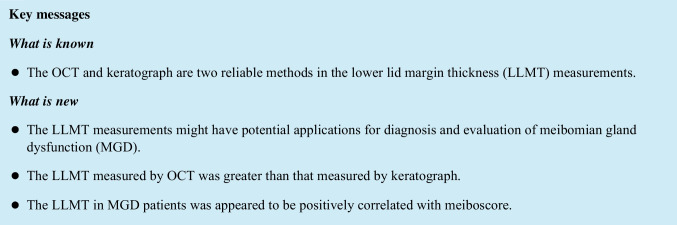


## Introduction

Meibomian gland dysfunction (MGD) is a clinically common ocular surface disease, which is closely related to evaporative dry eye [1]. A clinic-based patient cohort study in the European Union and the USA showed that 86% of dry eye patients demonstrated signs of MGD [2]. In Asia, the prevalence of MGD in dry eye subjects is also high, ranging from 46.2 to 69.3% [3–6]. Therefore, MGD-related evaporative dry eye is regarded as the most common form of dry eye disease (DED) [1], although the relevant evidence is not strong.

Population-based studies around the world have reported a prevalence of DED, with a range from approximately 5 to 50% [7]. The tear meniscus height (TMH) is a sensitive and important indicator in the diagnosis of DED [8–11], which can be measured by several different methods, such as slit-lamp examination with fluorescein staining, photography, video recording, meniscometry, Keeler Tearscope, optical coherence tomography (OCT), and keratograph [8–21]. Meanwhile, good repeatability and reproducibility of the TMH measurements with the OCT or keratograph have also been demonstrated in the previous literatures [17–21]. At present, OCT and keratograph are playing an increasingly important role in ophthalmology, which includes cornea, ocular surface, and DED [17–22].

It is well recognized that rounding and thickening of the lid margin are a common feature of MGD [23, 24], which had been regarded as one of the diagnostic indicators of MGD, but it is usually difficult to measure. At present, the changes of the lid margin thickness (LMT) are mainly based on individual clinician's judgment [24, 25]. Through the previous studies [26–29], we found that the LMT from the posterior lash line to the anterior edge of tear meniscus or Marx’s line/mucocutaneous junction (MCJ), i.e., the keratinized skin width, was a relatively constant feature of the lid margin and could be quantitatively measured by vernier micrometer, OCT, and keratograph.

Although several studies have assessed the agreement of TMH measurement between OCT and keratograph [19, 21], the existing literature lacks data on the repeatability and reproducibility of the lower lid margin thickness (LLMT) measurements between OCT and keratograph. Consequently, the purpose of this study was to investigate the intraobserver repeatability and diagnostic efficacy of the LLMT measurements performed by OCT and keratograph in adults with and without MGD and to assess the agreement between two devices.

## Material and methods

### Participants

This was a single-center, prospective, cross-sectional, observational study, which was an extension of our previous serial studies [26–29]. In this study, 108 volunteers aged 20 to 79 were recruited from the outpatient department of Longhua Hospital Affiliated to Shanghai University of Traditional Chinese Medicine, including 40 healthy participants and 68 MGD subjects. Only the data of right eyes were analyzed. The research followed the tenets of the Declaration of Helsinki and was approved by the Ethics Committee of Longhua Hospital Affiliated to Shanghai University of Traditional Chinese Medicine (No. 2021LCSY078). Written informed consent was obtained from all subjects after explaining the purpose of the study..

The diagnosis of MGD was established if a patient met the following criteria [23, 30]: (1) ocular symptoms; (2) abnormal morphologic lid margin features; (3) abnormal meibum quality and expressibility; (4) meibomian gland dropout; and (5) tear film lipid layer thickness. Patients with either items (1) + (2) or items (1) + (3) could be diagnosed as MGD. In addition, items (4) and/or (5) were used to enhance MGD diagnosis but were not mandatory.

The study exclusion criteria were [26–29] ocular surface disease Index (OSDI) scores ≤ 12 points; MGD patients were treated with topical artificial eyedrops 6 h before the examinations; irregular lid margin structures; history of chalazion or hordeolum within the past 3 months; topical anti-glaucoma therapy; ocular infection/inflammation; entropion and trichiasis; eyelid tumor; no eyelashes or aberrant eyelashes or central eyelashes loss; conjunctivochalasis; nystagmus; paralytic strabismus; worn contact lens within the past 3 months; had a history of intraocular surgery or ocular surgery; used isotretinoin (accutane) within the past 6 months; autoimmune disease requiring systemic treatment; and was pregnant or lactating women.

### Clinical assessments

Referring to our previous published articles [29, 30], all subjects were required to fill out the OSDI questionnaire and underwent the slit-lamp biomicroscopy examinations as well as clinical tests. For consistency, all clinical evaluations were made by a single specialist (X.-Q. L).

1. The fluorescein tear film break-up time (FBUT) was measured using sterile sodium fluorescein strips (Tianjin Jinming New Technology Development Co Ltd, Tianjin, China). FBUT was measured three times for each eye, and then the average was recorded [23, 30].
2. After FBUT measurements, corneal fluorescein staining (CFS) was observed under the slit-lamp biomicroscope with a cobalt blue filter. The cornea was split into 4 quadrants (superior, inferior, nasal, and temporal areas), and each quadrant was graded on a scale of 0 to 3: 0, no staining; 1, 1–30 dots; 2, > 30 dots without confluent patches; and 3, confluent patches, and/or filaments, and/or ulcer [23, 30].
3. After CFS, the Schirmer test without anesthesia (SIT) was observed with a sterile Schirmer strip (Tianjin Jinming New Technology Development Co Ltd), which was placed in the temporal one-third of the lower eyelid cul-de-sac of each eye for 5 min [23, 30].
4. The meibum quality grade scale of each of 8 glands in the central area of lower eyelid was 0 to 3 (0–24 points): 0, clear; 1, cloudy; 2, cloudy with granular; and 3, thick, like toothpaste [23, 30].
5. According to the number of 5 glands in the central third of lower eyelid from which the secretion could be expressed, the meibum expressibility was graded on a scale of 0 to 3: 0, 5 glands expressible; 1, 3 to 4 glands expressible; 2, 1 to 2 glands expressible; and 3, no glands expressible [23, 30].
6. The meibomian gland dropout grade (meiboscore) was observed by Keratograph 5 M (OCULUS Optikgerate GmbH, Wetzlar, Germany). The meiboscore of each eyelid was evaluated: 0 = normal; 1 = dropout ≤ 1/3; 2 = dropout > 1/3 and ≤ 2/3; and 3 = dropout > 2/3) [31].

### Intraoperator reproducibility

**Fig. 1:**
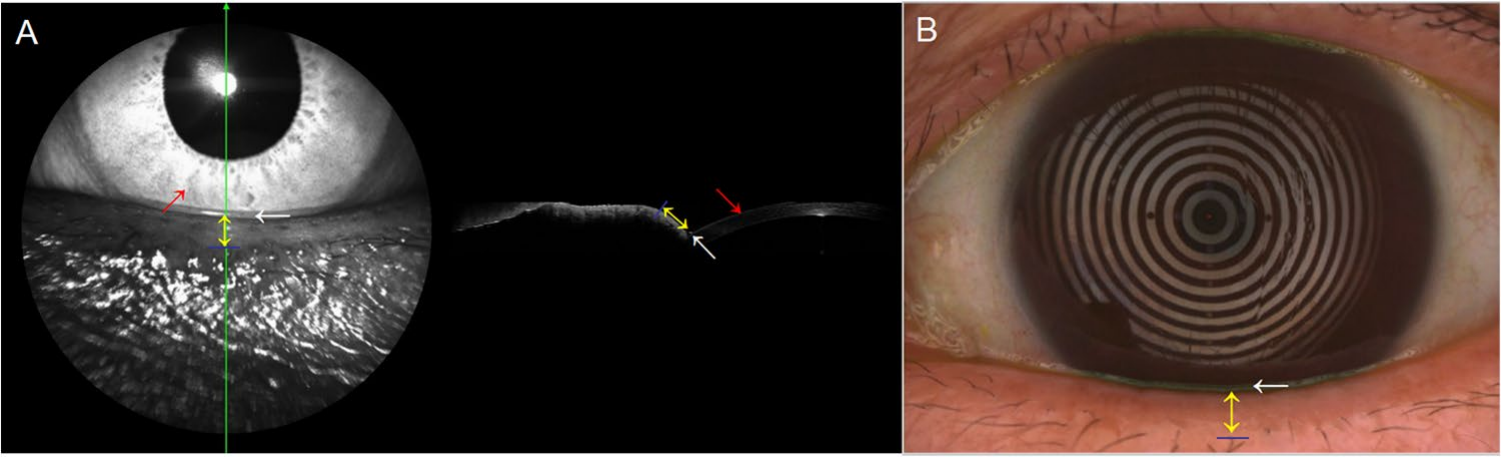
Measurement of the lower lid margin thickness (LLMT) with optical coherence tomography (OCT) and keratograph. **A** A representative OCT image. The long green arrow stands for the optimal position of OCT scanning; the two white arrows stand for the lower tear meniscus; the two red arrows stand for the cornea; the two blue lines stand for the posterior lash line; the two yellow double arrows stand for the LLMT (from the posterior lash line to anterior edge of the tear meniscus). **B** A representative keratograph image of the same eye. The blue line stands for the posterior eyelash line; the white arrow stands for the lower tear meniscus; the yellow double arrows stand for the LLMT

In this study, the LLMT was defined as the distance from the posterior lash line to the anterior edge of the tear meniscus at the central eyelid (Fig. 1) [26–29]. It referred to the keratinized lid margin surface width rather than the full thickness of lid margin. The temperature and humidity of the inspection room during all tests were maintained at 20 to 25 °C and 40% to 60%, respectively. Meanwhile, the ambient illuminations remained constant, and the surroundings were kept relatively quiet.

The LLMT measurements in 30 normal healthy volunteers were, respectively, performed by two well-trained operators (D.-H. W and X.-Q. L) with Spectralis OCT (Heidelberg Engineering GmbH, Heidelberg, Germany) and Keratograph 5 M (OCULUS Optikgerate GmbH, Wetzlar, Germany) under the same conditions, and the protocol process was basically the same as the measurement of TMH [17–21]. Operator 1 captured two consecutive representative images of the lower lid margin from each eye with each device; two additional consecutive images of the same eye were acquired by operator 2; then, the custom software were used to process the images, and the average value of 2 measurements for each eye was recorded as the LLMT. Furthermore, to avoid a subjective convergence of the results between two operators, the operators were blinded to each other’s results.

### Measurements of lower lid margin thickness

The lower lid margin thicknesses of 108 subjects (40 males and 68 females) were measured by the same examiner with OCT and keratograph. The protocol processes were basically the same as our previous researches [26–28]. In both normal and MGD subjects, two images from each eye were obtained by the same examiner (D.-H. W). The average value of two measurements was recorded as the LLMT. Furthermore, to avoid subjectively converging the results of two measurements, the first measurement data have been masked during the process of the second measurement after 2 weeks.

It should be emphasized that because of the rounded contour of lid margin and the poor tissue penetration of OCT and keragraph devices, it was difficult to measure the full thickness of lid margin including the mucosa. We all knew that the palpebral border of lid margin corresponded with the MCJ, i.e., the position of anterior edge of the tear meniscus. Since the boundary of MCJ was not very clear in the images, we regarded the anterior edge of tear meniscus to replace the MCJ as the posterior boundary of the keratinized lid margin skin in this study. In addition, the LLMT in the OCT and keragraph images was the line length rather than the actual arc length. At present, it was almost impossible to directly measure the arc length in the images. Therefore, in order to get an accurate measurement, we used two devices to measure the same area of lower lid margin from the posterior lash line to the anterior edge of tear meniscus, which had been proved to be a relatively constant feature of the lid margin [27, 28].

### Statistical analysis

All data were presented as the mean ± standard deviations (SD). The statistical analyses were performed with Student’s *t* test, nonparametric test, intraclass correlation coefficient (ICC), Bland–Altman plots, and Spearman’s rank correlation coefficient analysis. Receiver operating characteristic (ROC) curves with calculations of the area under the curve (AUC) were used to compare the diagnostic ability of LLMT measurements performed by OCT and keratograph for MGD. The total meiboscore of the upper and lower eyelids of both eyes was analyzed. *P* < 0.05 was considered significant. All analyses were performed with PASW Statistics version 25.0 (SPSS Inc., Chicago, IL, USA).

## Results

### Intraoperator reproducibility

**Fig. 2:**
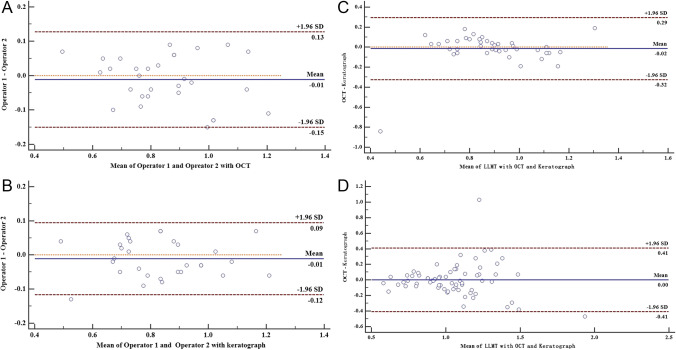
Bland–Altman plots. **A**, **B** Agreement between two operators in the LLMT measurements with OCT and keratograph. **C**, **D** Agreement between two devices in the LLMT measurements of normal and MGD subjects

The mean age was 39.1 ± 11.4 years in 30 healthy subjects (9 men and 21 women). The LLMT measured by two operators with OCT and keratograph were 0.85 ± 0.16 mm, 0.86 ± 0.18 mm, 0.83 ± 0.17 mm, and 0.84 ± 0.17 mm, respectively. Bland–Altman plots and ICC demonstrated a good agreement in the LLMT measurements with OCT and keratograph between two operators (ICC = 0.92 and 0.95; *P* =  < 0.001 and < 0.001, respectively) (Fig. 2). Therefore, using OCT and keratograph to measure the LLMT was judged to be reproducible.

### Measurements of lower lid margin thickness

The mean age of 40 normal subjects (18 males and 22 females) and 68 MGD subjects (23 males and 45 females) was 50.5 ± 14.2 years and 55.8 ± 15.5 years, respectively. The detailed demographic characteristics and clinical parameters of the study are presented in Tables 1 and 2, respectively.

**Table 1:** Demographic data for all participants

	Normal	MGD	*P* value
Number of subjects	40	68	_
Age, years			
Mean ± SD	50.5 ± 14.2	55.8 ± 15.5	0.07
Median	52.0	62.0	_
Minimum, maximum	23, 70	20, 78	_
Gender, *n* (%)			
Male	18 (45.0)	23 (33.8)	0.25
Female	22 (55.0)	45 (66.2)	

*MGD* meibomian gland dysfunction.

**Table 2:** Clinical parameter values for all subjects

Parameters	Normal	MGD	*P* value
OSDI	2.92 ± 2.88	42.41 ± 14.14	< 0.001
FBUT (s)	8.71 ± 2.41	2.87 ± 1.27	< 0.001
CFS	_	1.51 ± 2.13	NA
SIT (mm)	13.60 ± 5.21	8.09 ± 4.02	< 0.001
Meibum quality	_	9.09 ± 4.07	NA
Meibum expressibility	_	1.25 ± 0.79	NA
Meibography	0.68 ± 0.57	5.70 ± 2.55	< 0.001
TMH with keratograph (mm)	0.33 ± 0.05	0.26 ± 0.08	< 0.001
TMH with OCT (mm)	0.35 ± 0.07	0.29 ± 0.07	< 0.001
LLMT with keratograph (mm)	0.86 ± 0.16	1.03 ± 0.25	< 0.001
LLMT with OCT (mm)	0.90 ± 0.20	1.06 ± 0.27	0.003

*MGD* meibomian gland dysfunction, *OSD*I ocular surface disease index, *FBUT* fluorescein tear film break-up time, *CFS* corneal fluorescein staining, *SIT* Schirmer test without anesthesia, *TMH* tear meniscus height, *OCT* optical coherence tomography, *LLMT* lower lid margin thickness, *NA* not applicable.

**Fig. 3:**
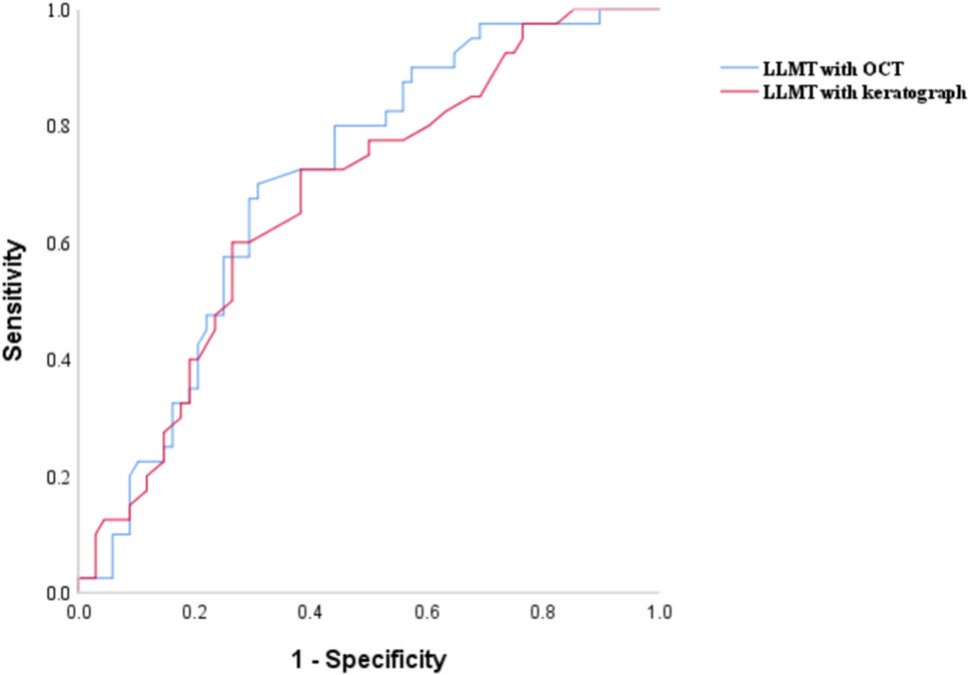
Comparison of the receiver operating characteristic (ROC) curves for the LLMT measurements using OCT and keratograph. The areas under two ROC curves were 0.71 and 0.68, respectively

**Fig. 4:**
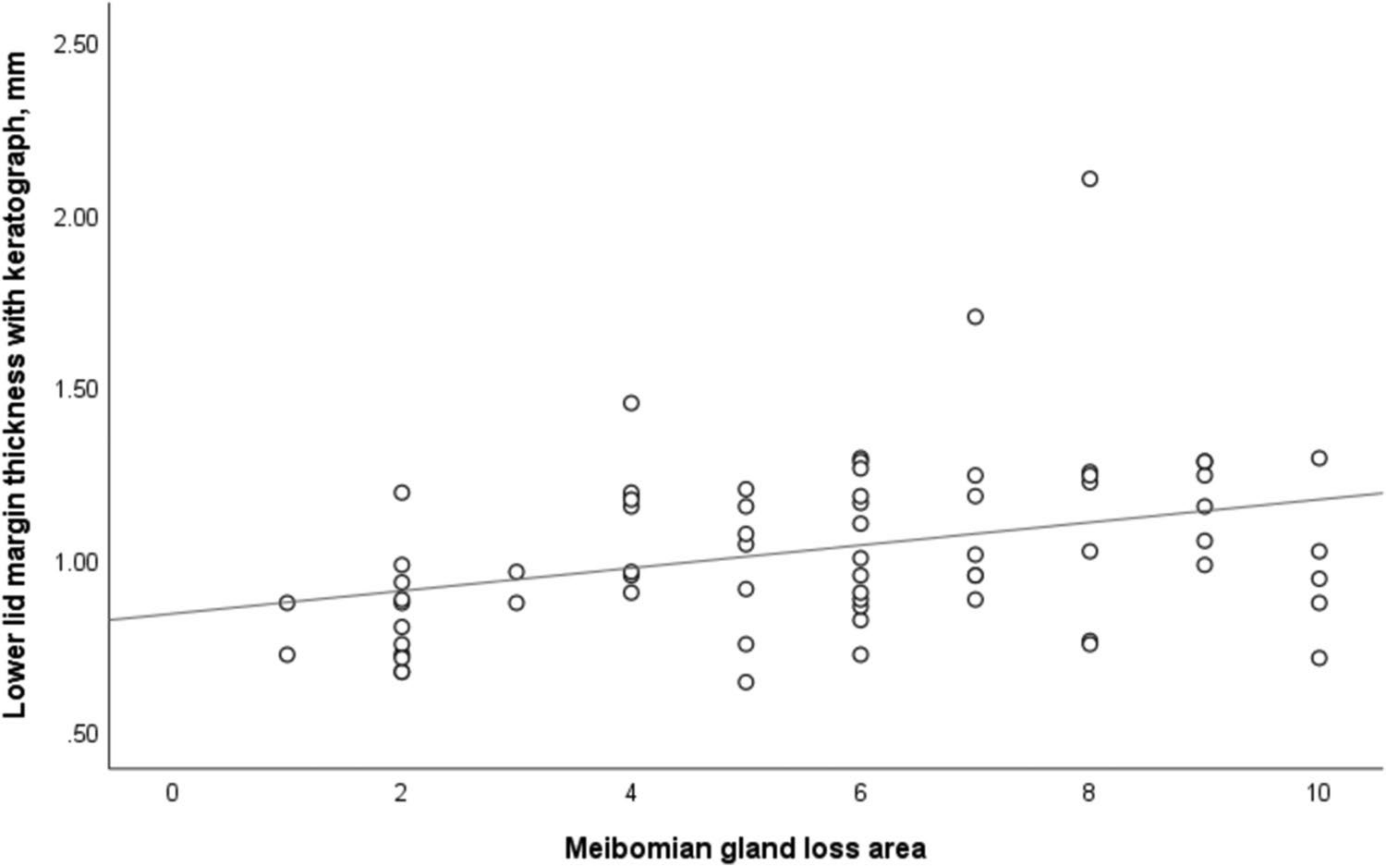
Association between the LLMT and meibomian gland loss area in MGD patients. The LLMT were slightly positively correlated with the extent of meibomian gland dropout (r_s_ = 0.37, *P* = 0.002)

The TMH measured by OCT and keratograph was significantly lower in MGD patients (0.29 ± 0.07 mm and 0.26 ± 0.08 mm, respectively) compared with normal subjects (0.35 ± 0.07 and 0.33 ± 0.05 mm, respectively). However, the LLMT measured by OCT and keratograph in MGD patients was significantly greater than that in normal subjects (1.06 ± 0.27 and 1.03 ± 0.25 mm vs. 0.90 ± 0.20 and 0.86 ± 0.16 mm, respectively). In both normal and MGD subjects, the TMH and LLMT measured by OCT were both greater than that measured by keratograph (*P* < 0.05), and ICC and Bland–Altman plots demonstrated good agreement in the LLMT measurements between two devices (ICC = 0.83 and 0.79, respectively) (Fig. 2). However, the correlation of TMH measurements between two devices in normal and MGD subjects was low (ICC = 0.63 and 0.53, respectively). The areas under two ROC curves of using OCT and keratograph to measure the LLMT of both normal and MGD subjects were 0.71 and 0.68, respectively (Fig. 3). Furthermore, the LLMT measured by keratograph in MGD patients appeared to be positively correlated with meiboscore (*r*_s_ = 0.37, *P* = 0.002) (Fig. 4).

In both normal and MGD subjects, the average age between the sexes was not significantly different (48.8 ± 13.7 vs. 51.9 ± 14.8 years, *P* = 0.45; 56.0 ± 17.4 vs. 55.2 ± 14.9 years, *P* = 0.56), and the LLMT measured by OCT and keratograph in males was about 0.1 mm thicker than that in females, but there seemed to be no significant difference (all *P* > 0.05). Additionally, the Spearman rank correlation coefficient demonstrated that the LLMT of both normal and MGD subjects measured by OCT and keratograph was significantly positively correlated with age (_normal_r_s_ = 0.76 and 0.61, *P* =  < 0.001 and < 0.001, respectively; _MGD_r_s_ = 0.59 and 0.44, *P* =  < 0.001 and < 0.001, respectively).

## Discussion

MGD is a chronic, diffuse abnormality of the meibomian glands, which is commonly characterized by obstructions of the meibomian gland terminal ducts and/or orifices, and alterations of the lipids of meibum [32]. In the advanced stage, this abnormality may be often accompanied by the rounding and thickening of the lid margin [23, 24], which is mainly subjectively assessed by the ophthalmologists at present [24, 25]. Therefore, objective evaluation of the lid margin is very valuable in the diagnosis of MGD.

Because of the rounded contour of eyelid margin, the LLMT is difficult to be measured. Despite all this, people are still working on it with several different methods, such as ruler, vernier micrometer, Scheimpflug camera, OCT, and keratograph [26–29, 33–35]. Recently, we found that compared with the traditional invasive measurement methods [26, 29, 33, 34], OCT and keratograph with good reproducibility and agreement were two non-contact, simple, and practical methods for quantitative evaluation of the LLMT [27, 28], which had a potential application for discriminating MGD patients from normal subjects [30].

The TMH measurements in DED have been proved to be useful and accurate [8–11, 36]. Good repeatability and reliability of OCT and keratograph for the TMH measurements have been investigated in previous reports [19, 21, 36], but the agreement between two devices was poor [19, 21, 36], and the TMH measured by keratograph tended to be lower than that measured by OCT [19, 21, 36]. In the present study, the TMH obtained with keratograph was lower than that obtained with OCT in both normal and MGD subjects, and the correlation of TMH measurements between two devices was also poor (ICC ≤ 0.63). Our findings were basically in line with the previous studies [19, 21, 36], and the image processing and operating principles between two devices could contribute to this difference.

In this pilot study, the LLMT measured by OCT and keratograph in MGD patients (1.06 ± 0.27 and 1.03 ± 0.25 mm) was significantly greater than that in normal subjects (0.90 ± 0.20 and 0.86 ± 0.16 mm), which was basically consistent with our previous findings [29], and further supported the theory that MGD might lead to thickening of the lid margin [23, 24]. Meanwhile, the LLMT measured by OCT was higher than that measured by keratograph in both normal and MGD subjects. Different algorithms for processing the image might contribute to this difference [19, 21], when an image was converted from the optical space into the physical space. Furthermore, our results were all lower than that reported in the previously published literatures [29, 33–35]. There might be several potential reasons for this discrepancy, such as different measurement positions (refer to the keratinized lid margin surface width in this study, excluding the mucosa and MCJ) and different measurement methods. However, the LLMT measurements between two devices showed a good agreement (ICC = 0.83 and 0.79, respectively), which suggested that the two methods can be substituted for each other in the LLMT measurements.

In addition, the AUC of the LLMT with OCT and keratograph is about 0.70, which suggested an approximately 70% chance that the ophthalmologists will correctly distinguish normal people from MGD patients through the LLMT measured by two devices. Since the lid margin changes in MGD were multifactorial, such as telangiectasia, dimpling or notching, irregularity, cicatrices, abnormal orifices, dilation of the meibomian gland ducts, and displacement of MCJ, in this study, to facilitate the LLMT measurements, we excluded MGD patients with irregular lid margin structures and did not investigate other factors, which might diminish the diagnostic efficacy of LLMT in distinguishing MGD subjects from healthy subjects.

In MGD subjects, we found that the LLMT had an approximately linear relationship with meiboscore, which further supported the observations that the lid margin abnormalities were associated with meibomian gland dropout [31, 37]. In addition, we found that the LLMT measured by OCT and keratograph changed with age, especially in normal subjects (*r*_s_ = 0.76 and 0.61, *P* =  < 0.001 and < 0.001, respectively), which was consistent with our previous observations [27–29]. However, Hykin and Bron [33] suggested that the lid margin thickened with age over the first 20 years of life, and the LMT in adults did not change with age. The possible explanation for this discrepancy was that the measurement tool and region were different.

Generally, a good agreement between two devices indicated that the LLMT measurements were reproducible. It will be of great value to investigate the LLMT with OCT and keratograph to discriminate MGD patients from normal subjects. In this study, since we excluded MGD patients with irregular lid margin structures, which might diminish the relevance of LLMT in MGD with age. In addition, exploring the relationship between the LLMT and the prognosis or severity of MGD will be the focus of our future work.

However, several limitations of our study should be noted. (1) This study only measured the keratinized lid margin surface width excluding mucosa, not full length of the LMT. (2) In view of the slope of upper lid margin and the presence of upper eyelashes, and the optical properties of OCT and keratograph devices, we only measured the LLMT and did not measure the upper LMT. (3) In addition, we excluded MGD patients with the irregular lid margin structures and did not compare the difference of LLMT between early-stage and advanced MGD. Further studies were warranted to provide more information.

## Conclusions

The OCT and keratograph were two rapid, noninvasive methods for assessing the LLMT with acceptable repeatability. The LLMT in MGD patients was greater than that in normal subjects and mildly positively correlated with meiboscore, which indicated that the LLMT measurements might have potential applications for the diagnosis and evaluation of MGD. Furthermore, we found that the LLMT measured by OCT was greater than that measured by keratograph.
